# Light-induced displacement of PLASTID MOVEMENT IMPAIRED1 precedes light-dependent chloroplast movements

**DOI:** 10.1093/plphys/kiac193

**Published:** 2022-04-27

**Authors:** Matthew E Dwyer, Roger P Hangarter

**Affiliations:** Department of Biology, Indiana University, Bloomington, Indiana, 47405, USA; Department of Biology, Indiana University, Bloomington, Indiana, 47405, USA

## Abstract

Light-dependent chloroplast movements are an actin-dependent cellular response to changes in the light environment that help plants maximize photosynthetic potential and reduce photodamage. Over a dozen proteins are known to be required for normal chloroplast movements, but the molecular mechanisms regulating the transformation of light perception into chloroplast motility are not fully understood. Here, we show that in Arabidopsis (*Arabidopsis thaliana*) the actin-bundling plasma membrane-associated proteins THRUMIN1, PLASTID MOVEMENT IMPAIRED1 (PMI1), and KINESIN-LIKE PROTEIN FOR ACTIN-BASED CHLOROPLAST MOVEMENT1 (KAC1) interact through the 14-3-3 proteins KAPPA and OMEGA. We also show that the interaction of PMI1 with 14-3-3 KAPPA and OMEGA is regulated by blue light activation of the Phototropin2 photoreceptor. Live-cell confocal microscopy revealed light-induced dynamic changes in the cellular localizations of PMI1 and KAC1. In particular, PMI1 was relocated away from irradiated areas of the plasma membrane in less than a minute after blue light exposure, consistent with PMI1 playing a critical role in initiating light-dependent chloroplast movements. We present a modified conceptual model for high light-dependent chloroplast movements in which PMI1 acts as the mobile signal that initiates a coordinated sequence of changes in protein–protein and protein–plasma membrane interactions that initiate the chloroplast movement response and determine where in the cell chloroplasts are able to anchor to the plasma membrane.

## Teaser

High light intensity-induced chloroplast movements in *Arabidopsis thaliana* mesophyll cells involves protein interaction changes and requires movement of the protein PMI1 from the plasma membrane.

## Introduction

As sessile organisms, plants have evolved mechanisms to acclimate to changes in resource availability in their environment. In particular, plants evolved the ability to reposition chloroplasts in leaf cells to most effectively capture the available light when light levels fluctuate as with the passage of shadows and sun flecks (Davis et al., 2011; Yamori, 2016; Gotoh et al., 2018). Under high-intensity blue light, chloroplasts in mesophyll cells position along the anticlinal sides of the cells, which protects chloroplasts from photodamage and improves light distribution to other cell layers (Kasahara et al., 2002; Li et al., 2009; Davis and Hangarter, 2012). Conversely, when light is limiting, chloroplasts reposition along the top and bottom cell faces in order to maximize light capture (Zurzycki, 1955). These dynamic processes occur on the timescale of minutes upon changes in light intensity. Chloroplast movements are driven by the blue light-specific photoreceptors, Phototropin1 (Phot1) and Phototropin2 (Phot2; Jarillo et al., 2001; Kagawa et al., 2001; Sakai et al., 2001). The Phototropins are light-dependent kinases that autophosphorylate upon stimulation and signal to downstream proteins (Christie et al., 1998; Briggs and Huala, 1999).

Dozens of proteins are known to be involved in normal chloroplast movement in Arabidopsis (*Arabidopsis thaliana*) but their molecular interactions and mechanisms remain elusive. In flowering plants, actin is essential for chloroplast movements (Malec et al., 1996; Kandasamy and Meagher, 1999; Kadota et al., 2009) but myosin motor proteins do not appear to play a role in chloroplast movement (Avisar et al., 2008). Light-induced changes in small chloroplast-associated actin (cp-actin) filaments have been shown to be required for chloroplasts movements (Kadota et al., 2009). Several chloroplast movement-associated proteins have been found to interact with F-actin and/or regulate cp-actin filament dynamics such as CHLOROPLAST UNUSUAL POSITIONING1 (CHUP1), which is an actin nucleator localized to the outer chloroplast membrane and is critical for the maintenance of the cp-actin filaments (Oikawa et al., 2003, 2008; Schmidt von Braun and Schleiff, 2008). THRUMIN1 is a light-dependent actin-associated protein that localizes to filamentous actin (Whippo et al., 2011) and in particular, cp-actin (Kong et al., 2013; Dwyer and Hangarter, 2021). Additionally, THRUMIN1 was shown to bundle F-actin in vitro and mutations in the *THRUMIN1* locus conferred severely hindered chloroplast movements in both the high and low light responses (Whippo et al., 2011). Under dark conditions, THRUMIN1 was found to uniformly associate with cp-actin around the chloroplast periphery and in response to blue light, the THRUMIN1-cp-actin reorganized to the forward-moving edge of the chloroplast (Kong et al., 2013; Dwyer and Hangarter, 2021).

Other proteins known to regulate actin dynamics in chloroplast movement are KINESIN-LIKE PROTEIN FOR ACTIN-BASED CHLOROPLAST MOVEMENT1 (KAC1) and its closely related protein, KAC2. The C-terminal domain of KAC1 has been shown to interact with F-actin in vitro and there was a reduction in the localization of cp-actin filaments around the chloroplast in *kac1* mutant plants (Suetsugu et al., 2010). Mutations in KAC1 hindered effective chloroplast movements but in the *kac1 kac2* double mutant, chloroplast movements were severely disrupted and lacked directionality or effectiveness (Suetsugu et al., 2010), although it was later shown that the chloroplasts could still move enough to change the light transmittance properties of whole leaves under stronger blue light (Suetsugu et al., 2016).

PLASTID MOVEMENT IMPAIRED1 (PMI1) is another protein critical for chloroplast movement (DeBlasio et al., 2005). Mutations in PMI1 were found to severely attenuate the low and high blue light chloroplast movement responses suggesting a major role for PMI1 in Phototropin signaling (DeBlasio et al., 2005). PMI1 has also been associated with regulation of cp-actin filaments at the chloroplast periphery (Suetsugu et al., 2015). PMI1 is a coiled-coil protein that contains a NT-type C2 domain (Zhang and Aravind, 2010). C2 domains are eukaryotic lipid-binding domains and display a wide range of lipid selectivity. The NT-type C2 domains have been shown to link actin/microfilament-binding adaptors to the membrane (Zhang and Aravind, 2010).

The Arabidopsis 14-3-3 LAMBDA isoform has been demonstrated to bind to Phot1 (Sullivan et al., 2009) and Phot2 (Tseng et al., 2012). The interaction of 14-3-3 LAMBDA with Phot2 was involved in regulating stomatal opening indicating a role for 14-3-3 proteins in regulating Phototropin-mediated responses (Tseng et al., 2012). 14-3-3 proteins are known for binding to phosphoserine and phosphothreonine-containing protein motifs and are capable of modulating the function of the interacting proteins (Jaspert et al., 2011). 14-3-3 proteins also form homo and heterodimers, and interactions with different combinations of 14-3-3 proteins have been shown to work combinatorically to confer the appropriate signal for a given environmental context (Jaspert et al., 2011). Recently, 14-3-3 proteins KAPPA and OMEGA were shown to associate with THRUMIN1 (Dwyer and Hangarter, 2021) further suggesting roles for 14-3-3 proteins in Phototropin signaling to downstream factors that drive chloroplast movement.

In this study, we show that THRUMIN1, KAC1, and PMI1 associate through a network of 14-3-3 protein associations. The interaction of PMI1 with 14-3-3 KAPPA and OMEGA was found to be greater in the light versus dark, whereas the interaction of 14-3-3 KAPPA and OMEGA with THRUMIN1 and KAC1 was not light dependent. We also found that PMI1 displayed rapid Phot2-dependent blue light-induced changes in its cellular localization and interaction with F-actin. Additionally, we observed light-induced changes in the localized F-actin-dependent clustering of KAC1. The time scale of the rapid light-induced changes in localization of both PMI1 and KAC1 indicate involvement in the onset of chloroplast movement. Thus, chloroplast movements appear to be regulated by light-dependent protein–protein interactions in a protein complex that contains at a minimum, THRUMIN1, PMI1, KAC1, and at least two 14-3-3 proteins. Our results also show that Phot2-dependent movement of PMI1 from the irradiated region of the plasma membrane and reorganization of plasma membrane-associated KAC1 protein islands precede chloroplast movements.

## Results

### Identification of THRUMIN1-interacting proteins

Proteins extracted from transgenic Arabidopsis leaves expressing THRUMIN1 fused with yellow fluorescent protein (THRUMIN1:YFP) were immunoprecipitated using anti-GFP (Green Fluorescent Protein) agarose beads compatible with YFP binding. The immunoprecipitated proteins were analyzed by mass spectrometry to identify candidate protein–protein associations. In samples prepared without protein crosslinking and with stringent washing of the beads, the only protein in the samples that appeared to be relevant to chloroplast movement was N-myristoyltransferase (AT5G57020.1). We suggest it is the enzyme responsible for myristoylation of THRUMIN1 since mutations in the predicted N-myristoylation site indicate it is essential for THRUMIN1 to function in chloroplast movement (Whippo et al., 2011; Supplemental Data Set 1). Additionally, 14-3-3 OMEGA has been demonstrated to appear in mass spectrometry submissions of THRUMIN1:YFP when no protein-crosslinker was used and associated with THRUMIN1 in a phosphorylation-dependent manner via in vivo co-immunoprecipitation (Co-IP) in *Nicotiana benthamiana* (Dwyer and Hangarter, 2021). When we immunoprecipitated THRUMIN1:YFP with formaldehyde crosslinking, several other proteins involved in chloroplast movements were found, including Phot2, PMI1, KAC1, various 14-3-3 proteins, and others of potential relevance (Table 1). The proteins in Table 1 represent a filtered list of candidates that had several unique peptides amongst three or more independent mass spectrometry submissions compared to a negative submission control of Arabidopsis plants expressing GFP:TUBULIN1 (Supplemental Data Set 1). No discernable differences in protein candidates were detectable by mass spectrometry in the crosslinked immunoprecipitations prepared from light- versus dark-treated leaves expressing THRUMIN1:YFP (Supplemental Data Set 1).

**Table 1:** Proteins found associated with THRUMIN1:YFP expressed in Arabidopsis noncrosslinked and crosslinked (1% formaldehyde) preparations

Gene Locus	Molecular Weight	Name
AT1G64500.1	41,016.8	Glutaredoxin family protein (THRUMIN1)
AT4G09000.1	29,931.8	General regulatory factor 1 (CHI)
AT1G78300.1	29,161.9	General regulatory factor 2 (OMEGA)
AT5G57020.1	49,799.4	Myristoyl-CoA:protein N-myristoyltransferase
AT5G58140.1	102,472.8	Phot2
AT1G09020.1	53,466.7	Sucrose nonfermenting-like protein
AT5G10470.1	141,036.8	KAC1
AT4G30160.1	109,328.3	Villin 4
AT5G67385.1	67,559.5	Phototropic-responsive NPH3 family protein (NCH1)
AT1G10200.1	21,040.3	GATA type zinc finger transcription factor family protein (WLIM1)
AT3G53420.1	30,474.5	Plasma membrane intrinsic protein 2A
AT3G26520.1	25,849	Tonoplast intrinsic protein 2
AT1G42550.1	93,875.8	PMI1
AT2G30520.1	65,854.8	Phototropic-responsive NPH3 family protein (RPT2)
AT3G55770.6	14,925.1	GATA type zinc finger transcription factor family protein (WLIM2b)

To validate the protein–protein associations with THRUMIN1 that we found by mass spectrometry, we further examined the associations via in vivo Co-IP assays in *N. benthamiana* leaves expressing THRUMIN1:YFP. We found that Phot2, PMI1, and KAC1 did not associate with THRUMIN1 (Supplemental Figure S1). However, we found in a previous study that THRUMIN1 interacts with 14-3-3 KAPPA and 14-3-3 OMEGA in *N. benthamiana* leaves (Dwyer and Hangarter, 2021). Although we also identified 14-3-3 CHI in our mass spectrometry results, Co-IP experiments with *N. benthamiana* leaves expressing THRUMIN1:YFP showed that 14-3-3 CHI did not associate with THRUMIN1:YFP when co-expressed in *N. benthamiana* leaves (Dwyer and Hangarter, 2021). Given that 14-3-3 proteins are known to heterodimerize (Chang et al., 2009), it is possible that CHI may heterodimerize to some extent with KAPPA and/or OMEGA proteins, which interact with THRUMIN1. The THRUMIN1-interacting proteins we identified by mass spectrometry in crosslinked preparations are likely to be a product of 14-3-3 scaffolding.

The 14-3-3 family is large but the direct association of KAPPA and OMEGA with THRUMIN1 appears to be specific to a subset of 14-3-3 proteins. In the 14-3-3 family, the LAMBDA isoform is the most closely related to KAPPA and OMEGA (Sullivan et al., 2009). LAMBDA has also been shown to interact with Phot2 and to function in blue light-induced stomata opening (Tseng et al., 2012). Co-IP experiments with LAMBDA showed that it also interacts with THRUMIN1, regardless of light treatment (Supplemental Figure S2A). KAPPA has also been shown via large-scale mass spectrometry affinity bait capture to associate with Phot2 (Shin et al., 2011) but under our conditions KAPPA, OMEGA, LAMBDA, AND CHI all failed to Co-IP with Phot2 (Supplemental Figure S2B).

Because we found that 14-3-3 OMEGA and KAPPA associate with THRUMIN1, we tested T-DNA insertion mutants of these loci for chloroplast movement defects. Whole leaf transmittance assays showed normal chloroplast movement phenotypes in the single and double mutant backgrounds (Supplemental Figure S3A), which is consistent with the functional redundancy of this group of 14-3-3 proteins. Previous work demonstrated that mutations to 14-3-3 LAMBDA also did not alter chloroplast movements (Tseng et al., 2012). Co-IP analyses of *N. benthamiana* leaf cells transiently expressing either THRUMIN1:YFP with KAPPA:HA or THRUMIN1:YFP with OMEGA:HA and subjected to darkness or 10 min of high-intensity blue light showed no evidence of light regulation of either 14-3-3 KAPPA or OMEGA association with THRUMIN1 (Supplemental Figure S3B).

### KAC1 associates with 14-3-3 OMEGA and KAPPA and coordinates with THRUMIN1 to facilitate chloroplast movement

**Figure 1:**
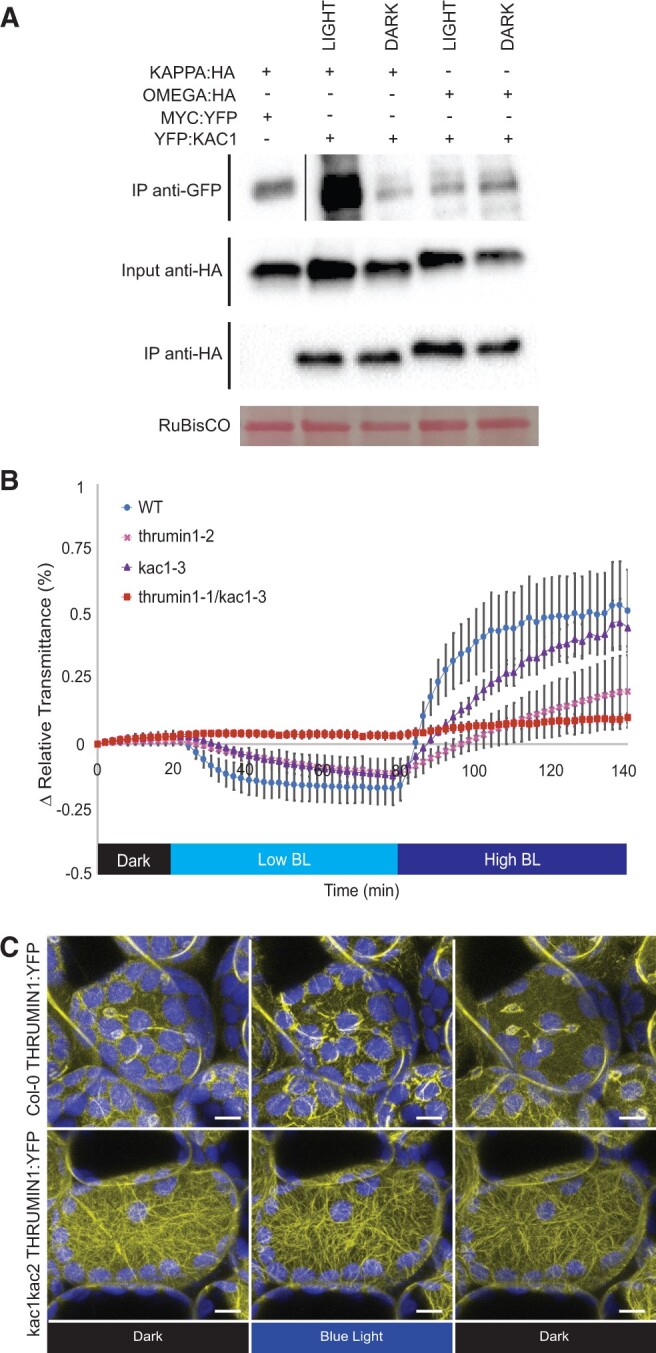
KAC1 associates with 14-3-3 KAPPA and OMEGA and works synergistically with THRUMIN1. A, 14-3-3 KAPPA and OMEGA co-immunoprecipitated with 35S_pro_:YFP:KAC1 co-transiently expressed with 35S_pro_:KAPPA:HA (*n* = 5) or 35S_pro_:OMEGA:HA (*n* = 5) in *N. benthamiana* leaves independent of blue light exposure. Prior to protein extraction, the leaves were exposed to either 10 min of high blue light (∼50 µmol m^−2^ s^−1^) or darkness 48-h postinfiltration. Protein bands do not represent true protein size since the blot images were stitched together. B, Leaf light transmittance assays with Col-0 wild-type, *thrumin1-2* mutant, *kac1-3* mutant, and *thrumin1-2 kac1-3* double mutant plants revealed a synergistic chloroplast movement phenotype with double mutant *thrumin1-2 kac1-3.* After establishment of the baseline level of leaf light transmittance after dark acclimation, chloroplast movement was induced by treatment with low blue light (BL, ∼2 µmol m^−2^s^−1^) followed by high blue light intensity (∼100 µmol m^−2^s^−1^). Error bars represent the standard deviation in transmittance values for 8–12 individual plants per genotype. The leaf transmittance assay was repeated three independent times for technical replicates. C, *kac1 kac2* double mutant plants expressing 35S_pro_:THRUMIN1:YFP displayed greater filamentous localization of THRUMIN1 in both dark and blue light-stimulated palisade mesophyll cells as compared to wild-type leaves (*n* = 6). Representative frames of dark (514 nm YFP excitation), blue light (470 nm), and post-blue light were selected. Chlorophyll autofluorescence is false-colored blue and YFP channel is in yellow. The scale bar = 5 µm.

Because 14-3-3 OMEGA and KAPPA were the only proteins, we found to associate with THRUMIN1 via Co-IP, we tested for their ability to associate with other protein candidates that were found by our crosslinked mass spectrometry analyses and found that YFP:KAC1 co-immunoprecipitated with both OMEGA:HA and KAPPA:HA (Figure 1A). KAC1 is a critical protein involved in chloroplast movement as indicated by defective chloroplast movement in *kac1* mutant plants (Suetsugu et al., 2010). We also found no discernable light/dark differences between KAC1 and 14-3-3 KAPPA or OMEGA interactions in *N. benthamiana* leaf cells expressing YFP:KAC1 with either KAPPA:HA or OMEGA:HA (Figure 1A). Because 14-3-3 LAMBDA is closely related to KAPPA and OMEGA, we also tested for interaction between LAMBDA and KAC1 but we only observed very weak and inconsistent evidence for interaction in both light/dark conditions (data not shown).

To better understand the relationship between KAC1 and THRUMIN1, we tested for chloroplast movement defects in *thrumin1-2 kac1-3* double mutant plants. The double mutant was more defective for both accumulation and avoidance responses as compared to the single mutants when assayed by changes in leaf light transmittance (Figure 1B), similarly to what was seen for the *thrumin1 kac1 kac2* triple mutant (Suetsugu et al., 2016). The synergistic phenotype of the *thrumin1 kac1* double mutant suggests they have independent functions in the blue light-induced avoidance response, but perhaps THRUMIN1 and KAC1 functions are coordinated through their interactions with 14-3-3 KAPPA and OMEGA, given the nature of 14-3-3 proteins in modulating pathways.

Because THRUMIN1 was found to localize to the actin cytoskeleton (Whippo et al., 2011) and KAC1 was found to be critical for the formation of cp-actin filaments (Suetsugu et al., 2010), we tested whether THRUMIN1 localization is dependent on KAC1. In *kac1 kac2* mutant Arabidopsis plants expressing the THRUMIN1:YFP transgene we found that instead of disrupting its localization to actin, THRUMIN1:YFP localized more robustly to F-actin in darkness and displayed an even more enhanced localization to cortical F-actin in response to blue light (Figure 1C; Supplemental Movies S1 and S2).

### KAC1 localizes to light-dependent clusters along the plasma membrane

**Figure 2:**
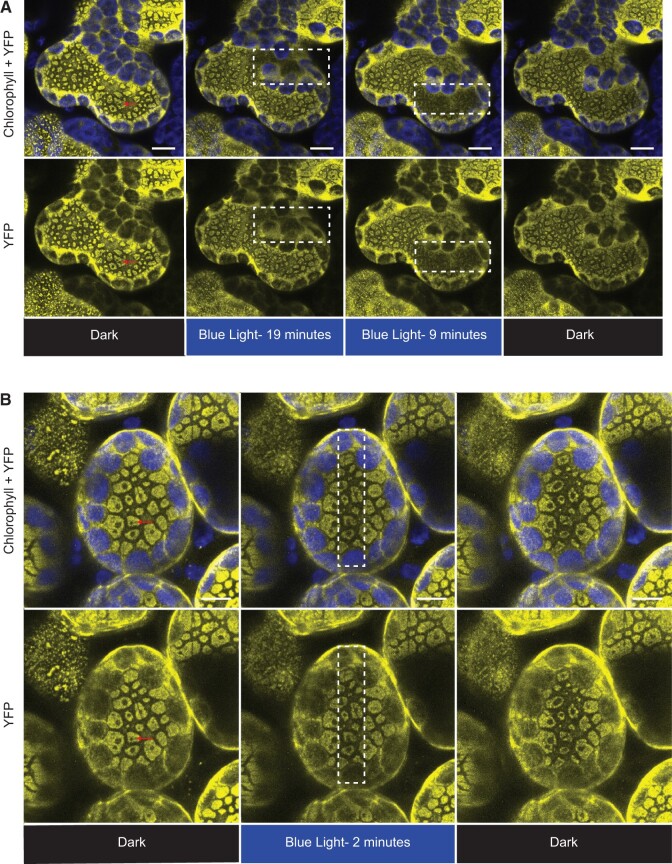
KAC1 localizes in blue light-sensitive clusters at the plasma membrane. A, Representative time-lapse frames from 35S_pro_:YFP:KAC1 transiently expressed in *N. benthamiana* mesophyll cells under dark (514 nm YFP excitation), blue light-treated (470 nm) and post blue light conditions. The 35S_pro_:YFP:KAC1 clusters (red arrows) were abundant in regions devoid of chloroplasts and dissipated in areas exposed to high blue light as the chloroplasts moved away. The clusters reformed after the blue light stimulus ended. See also Supplemental Movie S3. B, Latrunculin B (10 µM) inhibited the high blue light-stimulated reorganization of the KAC1 clusters (red arrows) (see Supplemental Movie S4). The cells were imaged continuously for 25 intervals and the blue light irradiated region is indicated by the white rectangles (representative frames depicted). Similar results were obtained in at least three independent experiments. Chlorophyll autofluorescence is false-colored blue and the YFP channel is separated. The scale bar = 5 µm.

In a previous study, transgenic KAC1 was found in both soluble and microsomal fractions and GFP fusions with KAC1 were found to be cytoplasmic (Suetsugu et al., 2010). We also saw KAC1 in the cytoplasm when we expressed C-terminal KAC1:YFP but a N-terminal YFP:KAC1 construct localized in clusters along the plasma membrane (Supplemental Figure S4). Time-lapse imaging showed the clusters had dynamic internal structure (Figure 2A). The clusters were primarily localized in areas lacking chloroplasts. However, YFP:KAC1 fluorescence was also observed between the chloroplast and plasma membrane, consistent with previous work that showed KAC1 is important for cp-actin filament regulation and attachment to the plasma membrane (Kadota et al., 2009; Suetsugu et al., 2010).

High intensity blue light microbeam stimulation resulted in dissipation of the YFP:KAC1 clusters and the YFP:KAC1 became localized in smaller puncta at the chloroplast periphery (Figure 2A;Supplemental Movie S3). When the blue light microbeam was terminated, the larger YFP:KAC1 clusters reformed and returned to regions of plasma membrane that were devoid of chloroplasts (Figure 2A;Supplemental Movie S3). The light-induced changes to the KAC1 clusters was actin-dependent since disruption of filamentous actin with latrunculin B prevented the blue light stimulation from inducing dramatic changes to the YFP:KAC1 clusters in *N. benthamiana* cells transiently expressing YFP:KAC1 (Figure 2B;Supplemental Movie S4).

### PMI1 associates with 14-3-3 OMEGA and KAPPA in a light-dependent manner

**Figure 3:**
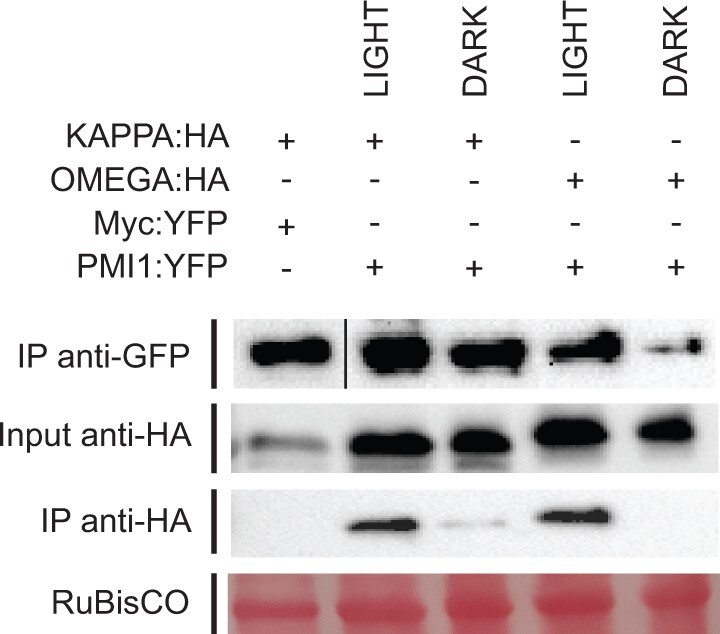
Interaction of 14-3-3 KAPPA and OMEGA with PMI1 is light-dependent. 35S_pro_:PMI1:YFP was co-transiently expressed with 35S_pro_:KAPPA:HA or 35S_pro_:OMEGA:HA in *N. benthamiana.* Prior to protein extraction for Co-IP analyses the leaves were exposed to either 10 min of high blue light (∼50 µmol m^−2^ s^−1^) or darkness 48-h postinfiltration. 35S_pro_:MYC:YFP was used as a negative control for all experiments and Ponceau-S stain was used as a loading control for total protein as demonstrated by RuBisCO. Protein bands do not represent true protein size since the blot images were stitched together. Similar results were obtained in at least three independent experiments.

To validate the protein association of PMI1 seen in our mass spectrometry results, PMI1:YFP was used as bait to identify which, if any, of our candidate proteins associate with PMI1. 14-3-3 OMEGA and KAPPA were found to associate with PMI1 via Co-IP, similar to KAC1 (Figure 3). When *N. benthamiana* leaves transiently expressing PMI1:YFP were harvested in darkness or under high blue light intensity before the Co-IPs were performed we found consistently less 14-3-3 OMEGA and KAPPA associated with PMI1:YFP in darkness compared to the light treatments (Figure 3). We also conducted Co-IP experiments with PMI1 and LAMBDA. On some blots, we saw evidence for weak interaction and on those blots, there appeared to be a slight increase in the light treatment. However, the interaction between PMI1 and LAMBDA could not be reliably reproduced (data not shown). Thus, while we found that THRUMIN1, KAC1, and PMI1 all associated with KAPPA and OMEGA, only PMI1 showed a light-dependent interaction.

### PMI1 undergoes rapid relocalization in response to high blue light stimulation of Phot2

**Figure 4:**
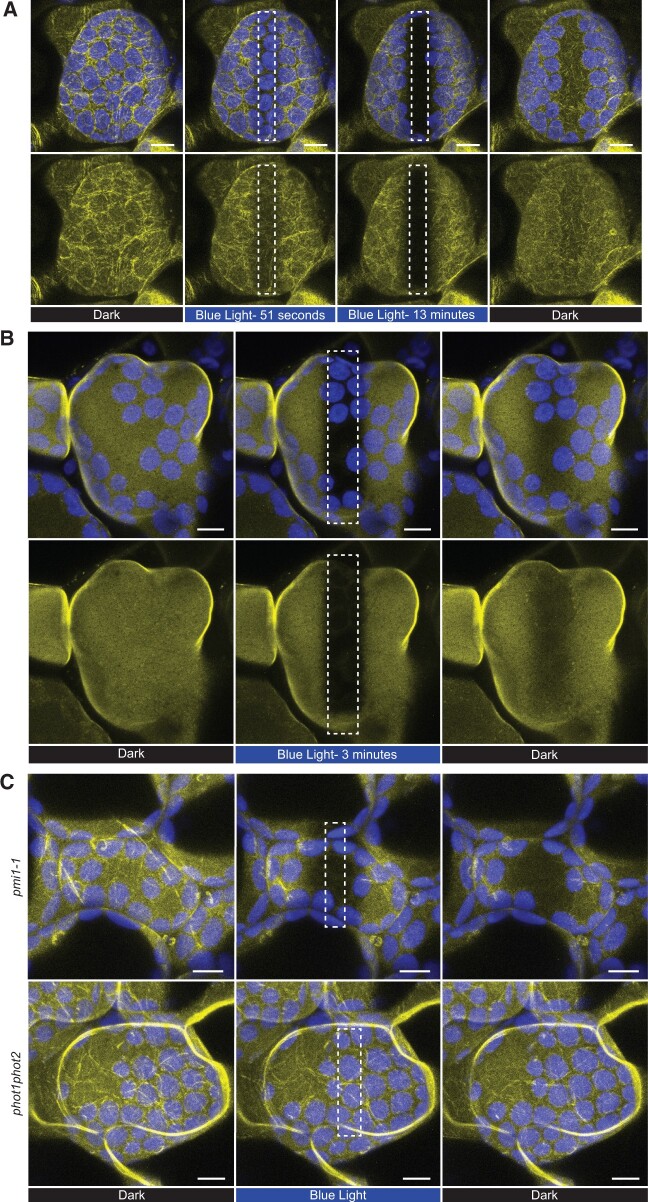
Blue light microbeam activation of Phot2 disrupts the interaction of PMI1 with cortical F-actin and repels PMI1 from the irradiated region of the plasma membrane. A, Time-lapse frames of *N. benthamiana* palisade mesophyll cells expressing 35S_pro_:PMI1:YFP showed that PMI1 localized to F-actin in darkness (514-nm YFP excitation) but rapidly left the F-actin and moved away from the region of high blue light stimulation (470 nm, white rectangle). PMI1 returned to the actin filaments after ending the blue light treatment (see Supplemental Movie S5). The results shown are representative of what was seen with 20 different cells in at least 15 independently transformed leaves. B, When F-actin was removed by treatment with latrunculin B, PMI1 was diffusely localized in dark conditions but when exposed to a high blue light microbeam (white rectangle), PMI1 still moved from the irradiated region (see Supplemental Movie S6). The results shown are representative of what was seen with seven different cells from at least five independently transformed leaves. C, Arabidopsis *pmi1-1* mutant plants stably expressing 35S_pro_:PMI1:YFP showed similar light-dependent dynamics while the relocalization response failed to occur in the *phot1 phot2* mutant background (see Supplemental Movie S7). Similar results were obtained in at least three independent experiments. Chlorophyll autofluorescence is false-colored blue and the YFP channel is false-colored yellow. The scale bar indicates a 5-µm distance.

We discovered that transiently expressed PMI1:YFP in *N. benthamiana* leaves was localized to cortical and cp-actin filaments after dark acclimation but when cells were exposed to high intensity blue light, PMI1:YFP rapidly left the actin filaments and dispersed from the blue light-exposed region (Figure 4A;Supplemental Movie S5). Similar results were observed in Arabidopsis cells stably transformed with PMI1:YFP (Figure 4C). PMI1:YFP became diffusely localized after disruption of F-actin with latrunculin B, but exposure to a blue light stimulus still resulted in a rapid loss of PMI1:YFP from the irradiated area (Figure 4B;Supplemental Movie S6). The rapid loss of PMI1:YFP from the blue light-treated area did not appear to be due to protein degradation as western blots/Co-IPs demonstrated equal levels of protein in dark versus light treatments (Supplemental Figure S5). Also, the blue light-induced relocalization of PMI1:YFP in the presence or absence of F-actin was completely blocked in *phot1 phot2* double mutant background and partially hindered in the *phot2* mutant background (Figure 4C;Supplemental Figure S6; Supplemental Movie S7), indicating that the relocalization of PMI1:YFP is primarily dependent on the Phot2 photoreceptor, which regulates the chloroplast avoidance response. The response to the high blue light microbeam was unaffected in the *phot1* mutant background as expected (Supplemental Figure S6). Our microscope laser is unable to provide a low light signal, so we were not able to determine if Phot1 alters PMI1:YFP localization.

**Figure 5:**
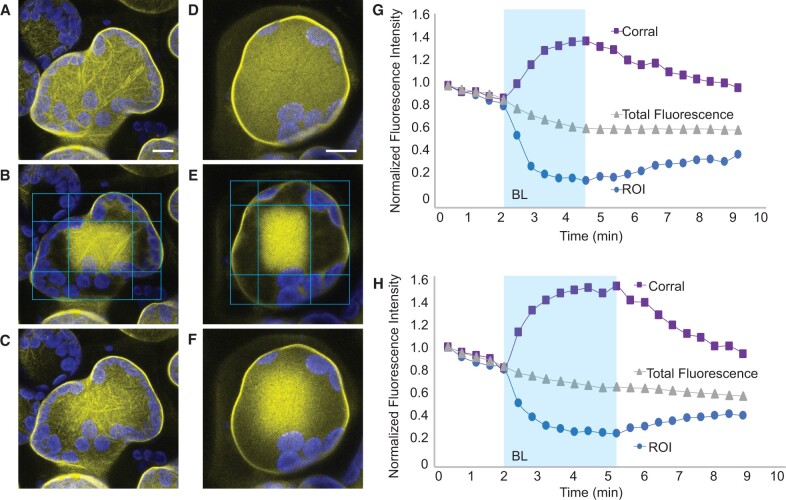
PMI1 is repelled from blue light-irradiated regions of cells to unirradiated regions in the presence or absence of F-actin. A, Confocal microscopy of transiently expressed 35S_pro_:PMI1:YFP in *N. benthamiana* palisade mesophyll leaf cells. B, When exposed to a rectangular perimeter of high blue light (BL) microbeams (outer rectangles), PMI1 rapidly moved away from the blue light irradiated areas and accumulated in the unirradiated region (center rectangle; representative micrograph). C, After removal of the blue light stimulus, PMI1 returned to the previously irradiated perimeter regions (see Supplemental Movie S8). D, E, and F, The same response occurred in leaf cells expressing 35S_pro_:PMI1:YFP that had been vacuum infiltrated with latrunculin B 1 h before imaging (see Supplemental Movie S9). G, Time course of normalized fluorescence intensity of PMI1:YFP quantified within the region of blue light (ROI) versus unirradiated corralled center. H, Time course of normalized PMI1:YFP fluorescence intensity after treatment with latrunculin B. In both the presence and absence of F-actin, the fluorescence increased inside the unirradiated corralled region in proportion to the YFP fluorescence decrease in the blue light-stimulated perimeter. The total average fluorescence intensity change showed little interference from YFP bleaching over the duration of the measurements. The average half-times for the blue light-induced increase in fluorescence in the corralled areas were 46.36 s without latrunculin B and 51.86 s with latrunculin B. Similar results were obtained in at least three independent microbeam corralling experiments. Scale bar in micrographs = 5 µm.

Because PMI1:YFP showed no signs of degradation in response to high blue light exposure, we wanted to better understand how PMI1 moves away from the blue light-treated region of the mesophyll cells. By exposing cells to a rectangular fence of blue light microbeams surrounding an unirradiated central corral, we observed that PMI1:YFP fluorescence increased inside the central corral while the areas exposed to blue light lost YFP fluorescence (Figure 5, A–C; Supplemental Movie S8). The same corralling response was seen in cells treated with latrunculin B (Figure 5, D–F; Supplemental Movie S9) indicating that PMI1 movement is not a result of cytoplasmic streaming or other actomyosin mechanisms. Time-lapse analysis of the changes in YFP fluorescence showed the average half-times for the blue light-induced increase in fluorescence in the corralled areas were 46.36 s (*n* = 3) without latrunculin B and 51.86 s (*n* = 3) with latrunculin B. Also, as long as the blue light microbeam was present, PMI1:YFP was prevented from returning to the irradiated area but when the blue light was removed, PMI1:YFP was able to diffuse back to the area it had been forced away from. Notably, although there was some bleaching of the PMI1:YFP fluorescence during the time course of these experiments, the total fluorescence level measured over the entire field of view remained relatively constant throughout the entire time course of these experiments. Thus, the loss of YFP fluorescence in the irradiated area was not the result of a decrease in the pH of the cytoplasm, which has been shown to diminish YFP fluorescence during acidification associated with programmed cell death (Young et al., 2010). The robustness and speed by which PMI1:YFP was seen to move away from the blue irradiated region and the similarly high rates of movement in both the presence or absence of F-actin demonstrate that blue light activation of Phot2 results in the development of what may be a potent and previously undetected repulsive force.

## Discussion

The work presented in this article shows that the three chloroplast movement proteins, THRUMIN1, KAC1, and PMI1, all associate in vivo with 14-3-3 KAPPA and 14-3-3 OMEGA (Figures 1A and 3; Supplemental Figure S3B). The association of THRUMIN1, KAC1, and PMI1 that we identified by mass spectrometry are likely to be transient since they were only observed when THRUMIN1:YFP was pulled down after protein crosslinking. However, the roles of 14-3-3 KAPPA and OMEGA are unclear since Arabidopsis encodes many structurally similar 14-3-3 isoforms that have redundant action and can form homo- and hetero-dimers (Tzivion et al., 2001; Chang et al., 2009; Johnson et al., 2010; Denison et al., 2011) and, accordingly, plants mutant for both genes retained normal chloroplast movements (Supplemental Figure S3A). Because THRUMIN1 does not appear to directly interact with KAC1 or PMI1 via Co-IP, our results suggest that these three proteins may interact via 14-3-3 KAPPA and OMEGA scaffolding. We also found that the interaction of PMI1 with 14-3-3 KAPPA and OMEGA was greater in the light versus dark (Figures 1, A and 3; Supplemental Figure S3B), which may indicate that light modulation of PMI1, THRUMIN1, and KAC1 interactions may be important for regulation of chloroplast movements.

Plants lacking PMI1 or KAC1 have both been shown to have reduced levels of cp-actin filaments when compared with wild-type suggesting that they are likely to be involved in stabilizing cp-actin (Suetsugu et al., 2010, 2015). Because THRUMIN1 colocalizes with cp-actin (Kong et al., 2013; Dwyer and Hangarter, 2021) and our protein interaction results suggest that THRUMIN1, KAC1, and PMI1 interact through 14-3-3 KAPPA and OMEGA scaffolding, we hypothesize that the in vivo THRUMIN1–cp-actin association is modulated by KAC1 and/or PMI1, possibly through their 14-3-3 interactions.

KAC1 has been shown to play a role in cp-actin filament regulation and cp-actin filaments are localized only at the interface between the chloroplast and the plasma membrane and not along the tonoplast side (Kadota et al., 2009; Suetsugu et al., 2010). It was also found that in the *kac1 kac2* double mutant background, chloroplasts displayed movements similar to cytoplasmic streaming in some cells indicating a major role for the KAC proteins in chloroplast attachment to the plasma membrane (Suetsugu et al., 2010). In addition, immunolocalization analysis in Arabidopsis root tips and confocal microscopy of tobacco (*Nicotiana tabacum*) BY-2 cells overexpressing GFP:KCA1 showed that KAC1 (renamed from KCA1) localized predominantly to the plasma membrane (Vanstraelen et al., 2006).

Our confocal microscopy of *N. benthamiana* cells expressing N-terminal tagged YFP:KAC1 showed that it localizes in large plasma membrane-associated clusters (red arrows), especially in areas lacking chloroplasts (Figure 2;Supplemental Figure S4). YFP:KAC1 fluorescence was also present between chloroplasts and the plasma membrane (Figure 2;Supplemental Figure S4). With blue light stimulation, the YFP:KAC1 clusters disassembled and YFP:KAC1 appeared to interact with the leading edge of chloroplasts as they moved in response to blue light stimulation (Figure 2A;Supplemental Movie S3). The YFP:KAC1 clusters we observed resemble protein-rich islands that have been characterized for a number of plasma membrane-associated proteins that partially rely on the actin cytoskeleton for function (Lillemeier et al., 2006). There is also precedence for protein islands of different composition to reorganize or combine clusters in response to an external cue or stimulus (Lillemeier et al., 2010). Our findings that KAC1 localizes to plasma membrane-associated protein islands along with the results of Vanstraelen et al. (2006), Kadota et al. (2009), and Suetsugu et al. (2010), support a role for KAC1 in anchoring chloroplasts to the plasma membrane via cp-actin. Our protein interaction experiments show that KAC1 and PMI1 indirectly interact with THRUMIN1. In addition, all three of these proteins have been shown to be required for the development or stabilization of cp-actin filaments (Suetsugu et al., 2010; Kong et al., 2013; Suetsugu et al., 2015). Thus, we suggest that scaffolding with 14-3-3 proteins provide the means for KAC1, PMI1, and THRUMIN1 to regulate plasma membrane anchoring and deanchoring of chloroplasts.

A striking finding from our investigations is the manner by which Phototropin activation caused PMI1 to move away from the blue light-irradiated region of palisade mesophyll cells. In the absence of high blue light stimulation, PMI1 was associated with actin filaments at or near the plasma membrane (Figure 4A;Supplemental Movie S5) but irradiation with high blue light microbeams caused PMI1:YFP to dissociate from actin filaments and to move away from the irradiated area where it remained until the blue microbeam was turned off (Figure 4A;Supplemental Movie S5). Our experiments with rectangular corrals made with high blue light microbeams demonstrated that as PMI1 leaves the irradiated areas it accumulates in the nonirradiated central corral both in the presence or absence of F-actin (Figure 5;Supplemental Movies S8 and S9). Notably, PMI1 remained outside the blue light-irradiated area as long as the blue light microbeam was on, even in the absence of F-actin. Our findings indicate that, continuous activation of the Phot2 photoreceptors is required to move PMI1 away from areas of the cell that are exposed to the high blue light stimulation (Figures 4 and 5;Supplemental Movies S5 and S8). The low light-induced chloroplast accumulation response (Tsuboi and Wada, 2010), requires continuous production of photoreceptor signals. The rapidity and extent of the exclusion of PMI1 from the blue light-irradiated areas, and the fact that the exclusion occurs in the absence of filamentous actin and, thus, in the absence of cytoplasmic streaming, suggests that the light stimulus results in the formation of some type of repulsive force that likely originates at the plasma membrane where the Phototropins reside (Sakamoto, 2002; Kong et al., 2006).

**Figure 6:**
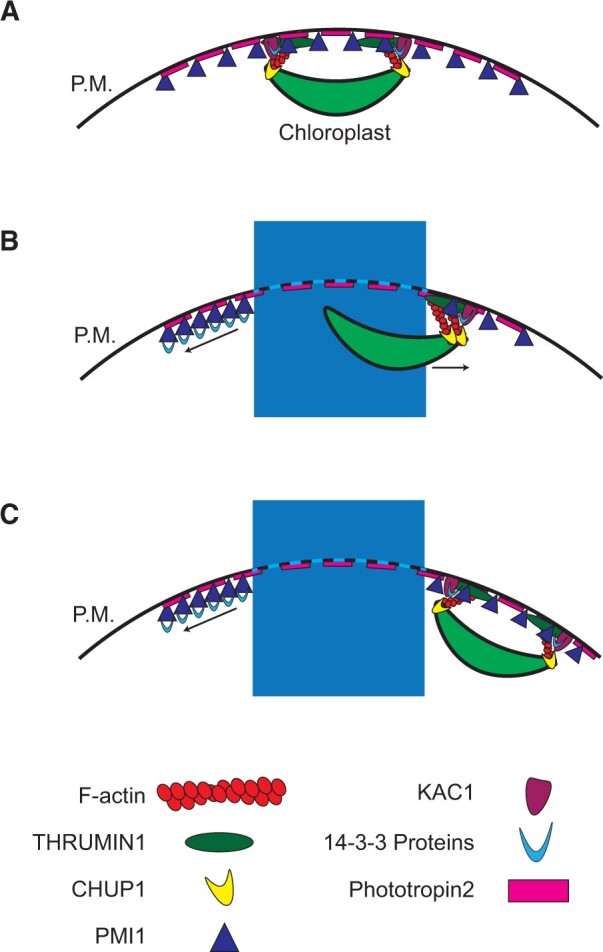
Conceptual model for chloroplast movements initiated by repositioning of PMI1 along the plasma membrane for proper anchorage by protein–protein associations. A, In darkness, THRUMIN1 and PMI1 localize to both cp-actin around the chloroplast periphery and to cortical F-actin. KAC1 is also important for the maintenance of cp-actin, and the 14-3-3 proteins KAPPA and OMEGA associate with THRUMIN1, KAC1, and PMI1. Phot2 is localized to the plasma membrane (P.M.), and CHUP1 is localized to the chloroplast membrane. B, In response to high blue light, Phot2 autophosphorylates and initiates the chloroplast movement response. PMI1 is rapidly repelled from the irradiated region, which is followed by the displacement of THRUMIN1 from F-actin, leading to de-anchoring of the chloroplasts. As PMI1 is cleared from the region exposed to high blue light it associates with 14-3-3 KAPPA and OMEGA more robustly. C, After leaving the irradiated area, PMI1 becomes localized along the unirradiated (or less irradiated) regions of the plasma membrane and THRUMIN1 reassociates to the leading edge of movement in conjunction with KAC1, which, along with THRUMIN1, are associated with 14-3-3 KAPPA and OMEGA regardless of blue light stimulation. According to this conceptual model, a gradient of blue light across a cell establishes where chloroplasts can reattach and anchor to the plasma membrane by the reassembly of PMI1-associated protein interactions. CHUP1 is an actin nucleator that generates cp-actin filaments that can bundle with THRUMIN1 at the plasma membrane. Net movement is thus achieved by the coordinated release of chloroplast from the brightest lit areas of the plasma membrane and reattachment to the less lit areas of plasma membrane in conjunction with the force generated by the growth of CHUP1-nucleated cp-actin filaments that push against the chloroplast outer envelope and THRUMIN1 in the plasma membrane.

The nature of the force, or mechanisms that moves PMI1 and keeps it away from the area receiving high blue light has not yet been determined. The plasma membrane-associated Phototropins have been shown to alter the membrane potential in plant cells, change the cytoplasmic pH by activation of plasma membrane H^+^-ATPase activity, and stimulate a rise in cytoplasmic Ca^2+^ and reactive oxygen species (Harada et al., 2003; Harada and Shimazaki, 2007; Majumdar and Kar, 2020). In addition, the inner plasma membrane of live eukaryotic cells is typically negatively charged as a result of the combined effects of the Nernst potential and negatively charged phospholipids that tend to be enriched in the inner leaf of the plasma membrane bilayer (e.g. phosphatidylserine, phosphatidic acid, phosphatidylethanolamine, and phosphatidylinositol species; Ma et al., 2017). Because the Phototropins are predominantly localized at the plasma membrane (Sakamoto, 2002; Kong et al., 2006) and undergo extensive phosphorylation when activated by blue light (Gallagher et al., 1988; Sakai et al., 2001; Boex-Fontvieille et al., 2014), phosphorylation of the Phototropins themselves may increase the negative charge near the membrane. PMI1 contains an N-terminal C2 domain, a protein motif often associated with anchoring sites for anchoring proteins to actin microfilament-based cytoskeletal scaffolds (Zhang and Aravind, 2010). C2 domains have also been implicated in binding to membrane lipids and calmodulin (Zhang and Aravind, 2010) and PMI1 becomes more phosphorylated in response to light (Boex-Fontvieille et al., 2014). Thus, we suggest that Phot2 activation may lead to localized changes to the properties of the plasma membrane as the primary first step in establishing the conditions to position PMI1, and possibly other, chloroplast movement proteins to the appropriate sites so their combined functions can bring about blue light-dependent chloroplast movements (Figure 6).

Current models for chloroplast movement are based on a motive force derived from the dynamics of cp-actin (Kadota et al., 2009; Kong and Wada, 2016). In brief, the CHUP1 protein, which is localized on the chloroplast outer envelope, polymerizes cp-actin filaments that extend toward the plasma membrane where cp-actin filaments bind to THRUMIN1. In the absence of blue light stimulation, CHUP1, THRUMIN1, and cp-actin distribute around the perimeter of the chloroplast outer envelope and the chloroplasts remain anchored in place. After blue light stimulation, cp-actin is severed (Kong et al., 2013) releasing the chloroplasts from the plasma membrane. THRUMIN1 and CHUP1 then relocate along the edge of the chloroplasts that are near regions where the light intensity tapers off. CHUP1 can then polymerize new cp-actin that extends to THRUMIN1 and the growth of new cp-actin filaments simultaneously push against the plasma membrane and against CHUP1 on the leading edge of the chloroplast to propel the chloroplasts away from the area of bright light (Kong et al., 2013; Kong and Wada, 2016). As the chloroplasts leave the high blue light-simulated region of the plasma membrane, CHUP1, THRUMIN1, and cp-actin reassemble around more of the perimeter of the chloroplast outer envelope until the chloroplasts exit the blue light stimulated regions and the cp-actin assembly again surrounds the entire chloroplast and effectively anchor the chloroplast by preventing biased movement.

Based on the observations made in this study, we propose a conceptual model (Figure 6) in which the repulsion of PMI1 from regions of a cell receiving high levels of blue light is a very early step in de-anchoring chloroplasts and suggest that the combination of cp-actin dynamics, cytoplasmic streaming, diffusion, and Brownian motion together contribute to the forces that move the chloroplasts. As unanchored chloroplasts enter unirradiated areas of the cell to where PMI1 was moved to, we further postulate that PMI1, in conjunction with KAC1, facilitates reattachment of chloroplasts to CHUP1-derived cp-actin as they exit areas of high blue light so cp-actin elongation can then push the chloroplast further along. As the chloroplasts move into the unirradiated areas, the cp-actin attachments eventually surround the periphery of the chloroplasts, which results in re-anchoring them in their new location (Figure 6). Microbial systems rely on diffusion to move proteins through the cell until they reach specific regions where they can localize by protein–protein interactions and/or protein–membrane charge (Laloux and Jacobs-Wagner, 2014). We propose that plant cells may be capable of rapidly modifying protein–membrane interactions over greater distances than needed in bacterial cells.

Although our work focused exclusively on the high blue light-induced chloroplast avoidance response, the repulsion of PMI1 that we discovered is likely to also be involved in the low blue light accumulation response since PMI1 is required for both responses (DeBlasio et al., 2005). In nature, leaf structure and cell shape result in light intensity gradients within leaf cells (Davis et al., 2011). Light gradients in the cells could therefore result in Phototropin-induced changes to the properties of the plasma membrane that generate gradients in the abundance of PMI1. We further suggest that light-induced forces generated by Phototropin at the plasma membrane are a key signal for initiating the high light chloroplast avoidance response and that the repositioning of PMI1 restructures the inner surface of the plasma membrane to control where anchoring of cp-actin can occur. Thus, the long-known actin-dependence on chloroplast movements is likely due to the combined actions of cp-actin filaments, cytoplasmic streaming, and the interactions of CHUP1, PMI1, THRUMIN1, KAC1, and other actin-binding chloroplast movement proteins. Perhaps other Phototropin-dependent repositioning of proteins at the plasma membrane, like the PIN-FORMED proteins (Zhou and Luo, 2018), may also be initiated by similar forces for their repositioning. Although the biochemical or biophysical mechanism that repositions PMI1 from one part of a cell to another is unknown, our findings indicate a need to better understand how the plasma membrane is remodeled in response to blue light stimulation before we can fully understand how plant cells can quickly and precisely reposition the proteins that move one of their largest organelles.

## Materials and methods

### Growth conditions

Arabidopsis (*A.* *thaliana*) and *N.* *benthamiana* seeds were surface sterilized with 19:1 87.5% (v/v) ethanol 30% H_2_O_2_ (v/v). After drying, the seeds were planted in Petri dishes on 1/2 MS (Mass Spectroscopy) salts and 0.6% (w/v) agar and cold stratified for 3 d at 4°C. The stratified seeds were then grown for 10 d under ∼100 µmol m^−2^s^−1^ white light in growth chambers with 12-h photoperiods at 23°C. The 10-d-old seedlings were then transplanted to fertilized (20-20-20) potting mix (PromixB) and grown under ∼120 µmol m^−2^s^−1^ white light and a 12-h photoperiod. The same process was used for transgenic lines except the 1/2 MS media contained 30 mg/mL BASTA (glufosinate ammonium; Cayman Chemical) for selection of the transgenic plants.

### Cloning

All genomic gene products were cloned into the entry Gateway vector pBSDONR P1P4 backbone (Qi et al., 2012) using the primers listed. Error-free sequences were recombined into the pEG100 plant expression vector (Earley et al., 2006) with a pBSDONR P4rP2 YFP clone using LR Clonase II (Invitrogen, Carlsbad, CA, USA) to create a final destination vector to be transformed into *Agrobacterium tumefaciens* strain GV3101. For the 35S_pro_:YFP:KAC1 construct, YFP was inserted in the pBSDONR P1P4 backbone and KAC1 was inserted in the pBSDONR P4rP2 backbone to create the N-terminally tagged version using the same Gateway recombination method. The gene fusions were then transformed into Arabidopsis Col-0, *thrumin1-2* (SALK_027277), *pmi1-1*, *phot1-1*, *phot2* (SALK_142275), *phot1-1 phot2*, and *kac1-3 kac2-1* backgrounds using the *Agrobacterium-*mediated floral dip transformation method (Clough and Bent, 1998).

### *Agrobacterium*-mediated transient expression

*Agrobacterium* *tumefaciens* (strain GV3101) carrying the different gene constructs in the pEG100 plant expression vector were cultured in LB media and resuspended in 10-mM MgCl_2_ 10-mM MES pH 5.6 to an optical density (OD)_600_ of 0.2. The culture was incubated for several hours with 3′,5′-Dimethoxy-4′-hydroxyacetophenone (Acetosyringone) to induce virulence and then injected into *N. benthamiana* leaves. After 48 h of incubation, leaves were excised and mounted for imaging the fluorescence of the expressed gene products on a Leica SP8 scanning confocal microscope using imaging parameters as described below.

### Live-cell confocal microscopy

Prior to mounting leaf samples on slides, the plants were low-light acclimated for ∼3 h under ∼10 µmol m^−2^s^−1^ light intensity to facilitate arrangement of the chloroplasts on the periclinal cell face before imaging. After low-light acclimation, small leaf sections excised and mounted for imaging and incubated in Perfluoroperhydrophenanthrene (CAS Number 306-91-2; Millipore/Sigma Burlington, MA, USA) to clear out the air spaces and optimize image resolution. All imaging was acquired using a Leica SP8 Scanning Confocal microscope with an inverted 40×/1.10 water objective lens. During the first 5 min, time-lapse images of YFP fluorescence (emission collected at 525–600 nm) in palisade mesophyll cells were captured at continuous intervals of ∼25 s with only YFP excitation using 5% (minimal gain) 514-nm laser illumination to prevent activation of the Phototropin photoreceptors. The samples were then exposed for ∼15 min with 1% whole-field or microbeam blue light stimulation (470 nm) to induce the chloroplast avoidance response while imaging YFP fluorescence with 514 nm excitation. The 470-nm blue light microbeam provided 50–100 nW of continuous power during the treatment as measured from the objective lens output with a Thorlabs Power Meter (PM100). Preliminary tests showed this was sufficient power to induce a strong high blue light response with minimal YFP photobleaching. The blue light treatment was then stopped, and the cells were imaged for an additional ∼5–10 min with 514-nm YFP excitation in the absence of blue light stimulation. Throughout the imaging process, the 514-nm excitation beam provided about 1 µW of power, and chlorophyll emission was collected at 650–720 nm. In all cases, the top 10–12 µm of the palisade mesophyll cell was imaged in 0.42 µm Z-steps. The images were combined by Z-projection and analyzed using FIJI software.

To inhibit F-actin formation, excised leaf sections were vacuum infiltrated with 10-µM Latrunculin B (Millipore/Sigma; L5288) and incubated for 1 h before imaging with the parameters listed above. The lack of chloroplast and cytoplasmic movements served as a control for drug functionality.

To create a corralled region of PMI1:YFP fluorescence, 4 rectangular blue light (470 nm) microbeams were placed around the perimeter of a palisade mesophyll cell and imaged as described above. FIJI software was used to calculate the average fluorescence intensities over a ∼10-micron line scan in an area within the microbeam and within the corralled region over each time interval. The averaged fluorescence intensities from three independent experiments were plotted over time relative to the starting fluorescence intensity point. Whole-cell average fluorescence intensity was also calculated over time using FIJI line tools to accommodate for photobleaching. The rate of PMI1 translocation in response to blue light was assessed by dividing the summed intensity of the corral region into the summed intensity of the entire cell after subtracting the camera offset. The resulting change in the fraction of total fluorescence in the corral was fit to an exponential curve in plants with, or without, latrunculin B treatment.

### Mass spectrometry

To identify protein–protein associations, ∼3.0 g of rosettes excised from transgenic Arabidopsis plants expressing THRUMIN1:YFP were exposed to high-intensity blue light for 10 min. The rosettes were then immediately vacuum infiltrated for protein crosslinking in 1.0% (v/v) formaldehyde solution containing 1.0-M Tris–HCl pH 7.5, 0.5-M EDTA, 400-mM sucrose, and 200-mM phenylmethylsulfonyl fluoride (PMSF). The rosettes were then rinsed with cold diH_2_O, blotted dry, flash frozen in liquid nitrogen and pulverized. The powdered plant material was mixed with lysis buffer containing 50-mM Tris–HCl pH 7.5, 150-mM NaCl, 10% (v/v) Glycerol, 1-mM EDTA, 1% (v/v) NP40, and plant protease inhibitor cocktail tablets (Millipore/Sigma; cOmplete, Mini, EDTA-free Protease Inhibitor Cocktail; 11836170001) and mixed for 30 min on a tube rotator at 4°C. The plant lysate was then centrifuged at 10,000*g* and the supernatant was added to washed GFP-Trap agarose beads (gta20; ChromoTek, Munich, Germany.) for rotation at 4°C for 3 h. After incubation with the lysate, the beads were washed 5 times with the lysis buffer at 4°C with 1,000*g* centrifugation to pellet the beads between washes. The washed beads were then suspended in a 1× sodium dodecyl sulfate (SDS)-loading buffer and boiled at 95°C for 15 min to reverse the crosslinking. Samples were centrifuged at a low speed (1,000*g*) and the supernatant was loaded into a Mini-PROTEAN TGX 4%–20% (w/v) gradient gel (Bio-Rad, Hercules, CA, USA). The samples were migrated enough for the proteins to enter the gel and excised as a single band to be submitted to the Indiana University Laboratory for Biological Mass Spectrometry facility for analysis.

For mass spectrometry analysis, tryptic peptides were injected into an Easy-nLC 100 high performance liquid chromatography (HPLC) system coupled to an Orbitrap Fusion Lumosmass spectrometer (Thermo Fisher Scientific, Waltham, MA, USA). Specifically, peptide samples were loaded onto an Acclaim PepMap 100 C18 trap column (75 μm × 2 cm, 3-μm bead size with 100 Å pores) in 0.1% (v/v) formic acid. The peptides were separated using an Acclaim PepMap RSLC C18 analytical column (75 μm × 25 cm, 2-μm bead size with 100 Å pores) using an acetonitrile-based gradient (solvent A: 0% [v/v] acetonitrile and 0.1% [v/v] formic acid; solvent B: 80% [v/v] acetonitrile and 0.1% [v/v] formic acid) at a flow rate of 300 nL/min. A 30-min gradient was performed as follows: 0–0.5 min, 2%–8% B; 0.5–24 min, 8%–40% B; 24–26 min, 40%–100% B; 26–30 min, 100% B, followed by re-equilibration to 2% B. Electrospray ionization was then performed with a nanoESI source at a 275°C capillary temperature and 1.9-kV spray voltage. The mass spectrometer was operated in data-dependent acquisition mode with mass range of 400–2,000 *m/z*. Precursor ions were selected for tandem mass spectrometry analysis in the Orbitrap with 3-s cycle time using higher energy collisional dissociation at 28% collision energy. The intensity threshold was set at 5 × 10^4^. The dynamic exclusion was set with a repeat count of 1 and exclusion duration of 30 s. The resulting data were searched in Protein Prospector (http://prospector.ucsf.edu/ prospector/mshome.htm) against the Arabidopsis database. Carbamidomethylation of Cys residues was set as a fixed modification. Protein N-terminal acetylation, oxidation of Met, protein N-terminal Met loss, pyroglutamine formation, phosphorylation on STY were set as variable modifications. In total, three variable modifications were allowed. Trypsin digestion specificity with one missed cleavage was allowed. The mass tolerance for precursor and fragment ions was set to 10 ppm for both. Peptide and protein identification cutoff scores were set to 15 and 22, respectively.

### Co-IP assays

For Co-IP assays, samples were extracted from ∼0.5 g of *N. benthamiana* leaves that were transiently expressing the transgenes of interest using the same protocol used for mass spectrometry but without crosslinking. All of the transgenes were driven by the viral 35S promoter. Co-IP’s were conducted with samples prepared from leaves 48-h postinfiltration. When noted, the leaves were subjected to either 10 min of high blue light (∼50 µmol m^−2^ s^−1^) or darkness before extraction. 35S_pro_:MYC:YFP was used as a negative control for all experiments. Ponceau-S stain was used as a loading control for total protein as demonstrated by RuBisCO levels.

**Table:** 

Primer list
THRUMIN1 attB1 FWD	GGGGACAAGTTTGTACAAAAAAGCAGGCTTAATGGGGTGTACATCTTCCAAG
THRUMIN1 attB4 REV	GGGGACAACTTTGTATAGAAAAGTTGGGTGATTAACAAAACACACGGGACAACG
PHOT2 attB1 FWD	GGGGACAAGTTTGTACAAAAAAGCAGGCTTAATGGAGAGGCCAAGAG
PHOT2 attB4 REV	GGGGACAACTTTGTATAGAAAAGTTGGGTGGAAGAGGTCAATGTCC
14-3-3 KAPPA attB1 FWD	GGGGACAAGTTTGTACAAAAAAGCAGGCTTAATGGCGACGACCTTAAGC
14-3-3 KAPPA attB4 REV	GGGGACAACTTTGTATAGAAAAGTTGGGTGGGCCTCATCCATCTGCTCC
14-3-3 OMEGA attB1 FWD	GGGGACAAGTTTGTACAAAAAAGCAGGCTTAATGGCGTCTGGGCGTGAAG
14-3-3 OMEGA attB4 REV	GGGGACAACTTTGTATAGAAAAGTTGGGTGCTGCTGTTCCTCGGTCGGT
14-3-3 CHI attB1 FWD	GGGGACAAGTTTGTACAAAAAAGCAGGCTTAATGGCGACACCAGGAGC
14-3-3 CHI attB4 REV	GGGGACAACTTTGTATAGAAAAGTTGGGTGGGATTGTTGCTCGTCAGCGGGT
KAC1 attB1 FWD	GGGGACAAGTTTGTACAAAAAAGCAGGCTTAATGGCCGATCAGAGAAGTAAAAC
KAC1 attB4 REV	GGGGACAACTTTGTATAGAAAAGTTGGGTGCTCCAGTTCACTAACAAGGTCCT
PMI1 attB1 FWD	GGGGACAAGTTTGTACAAAAAAGCAGGCTTAATGGCAGGAGAATATTCCG
PMI1 attB4 REV	GGGGACAACTTTGTATAGAAAAGTTGGGTGATGCAATTTCACATCAGGG
KAC1 attB4r FWD	GGGGACAACTTTTCTATACAAAGTTGCGATGGCCGATCAGAGAAGTAAAAC
KAC1 attB2 REV	GGGGACCACTTTGTACAAGAAAGCTGGGTATTACTCCAGTTCACTAACAAG

For the YFP:KAC1 IP experiments, samples were denatured in 2.5× SDS loading buffer and heated at 50°C for 10 min to prevent protein aggregation. All other proteins followed the standard boiling/sodium dodecyl sulfate–polyacrylamide gel electrophoresis (SDS–PAGE) protocol (described above). After SDS–PAGE, the protein was transferred to a nitrocellulose membrane (GE Lifescience Product #10600003) and stained with Ponceau-S for validation of protein transfer. The blots were then blocked in 5% (w/v) skim milk for at least 1 h. For GFP/YFP detection, primary anti-mouse GFP antibody (Novus Biological; NB600-597) was applied at 1:7,500 dilution and incubated overnight at 4°C with horizontal platform shaking (62 RPM) followed by incubation with secondary goat anti-mouse-HRP antibody (A-10668; Invitrogen Waltham, MA, USA) with three 5-min Tris-buffered saline 0.1% (v/v) Tween 20 washes in-between and after antibody applications. For HA detection, anti-HA-HRP (3F10; Sigma, St Louis, MO, USA) conjugated antibody was applied at 1:7,500 dilution for 1 h at room temperature. Blots were incubated with Clarity Western ECL Substrate (#1705061; Bio-Rad) for 5 min and imaged using a ChemiDoc Imaging System to detect chemiluminescence of the blotted proteins.

### Leaf light transmittance chloroplast movement assay

Leaf discs (7 mm) were made with a hole punch and placed on a 0.5% (w/v) agar pad in wells of clear-bottom 96-well plate (Falcon) sealed with Microseal “A” film (Bio-Rad). The film was punctured over each well with a needle to allow for gas exchange. The prepared plates were dark-acclimated for a minimum of 6 h before placement in a BioTek Cytation 3 Imaging Reader. The baseline level of light transmittance through the leaf discs was calculated from measurements of absorbance values of 660-nm red light taken every 2 min for 20 min (red light does not activate chloroplast movement). To induce chloroplast movement in the cells, the plate reader was programmed to eject the plate for exposure to the selected light intensity for 2 min. The plate was then moved back into the plate reader for recording of transmittance values (660-nm red light absorbance) for each well. After each reading, the plate was re-ejected to return to the blue light treatment. The cycle of recording transmittance values and incubating with blue light was repeated for the indicated time periods for a given light treatment. The calculated changes in light transmittance values were normalized to be relative to the starting “dark” position values.

### Accession numbers

Sequence data from this article can be found in the GenBank/EMBL data libraries under accession numbers THRUMIN1 (At1G64500), 14-3-3 KAPPA (AT5G65430), 14-3-3 OMEGA (AT1G78300), Phot2 (AT5G58140), PMI1 (AT1G42550), and KINESIN-LIKE PROTEIN FOR ACTIN BASED CHLOROPLAST MOVEMENT1 (AT5G10470).

## Supplemental data 

The following materials are available in the online version of this article.

**Supplemental Figure S1.** THRUMIN1 does not directly associate with Phot2, PMI1, and KAC1.

**Supplemental Figure S2.** 14-3-3 LAMBDA associates with THRUMIN1 regardless of light treatment, but not Phot2.

**Supplemental Figure S3.** THRUMIN1 associates with 14-3-3 KAPPA and OMEGA independent of light stimulus, but *kappa* and *omega* mutants have normal chloroplast movements.

**Supplemental Figure S4.** Arrangement of the YFP fusion protein affects the localization of KAC1.

**Supplemental Figure S5.** The PMI1 relocalization from blue light-irradiated regions of the cell is not due to protein degradation.

**Supplemental Figure S6.** Phot2 is the main photoreceptor initiating the relocalization of PMI1 in high blue light.

**Supplemental Movie S1.** 35S_pro_:THRUMIN1:YFP expressed in the Col-0 wild-type background with whole-field blue light (470 nm) treatment demonstrated the biased reorganization of the THRUMIN1-cp-actin filaments on the leading edge of the chloroplast during movement.

**Supplemental Movie S2.** 35S_pro_:THRUMIN1:YFP expressed in the *kac1-3 kac2-1* double mutant background with whole-field blue light (470 nm) treatment elicited a robust hyper-localization of THRUMIN1 to the cp-actin and cortical actin filaments.

**Supplemental Movie S3.** Transient expression of 35S_pro_:YFP:KAC1 in *N. benthamiana* exposed to regions of blue light (rectangles) demonstrated protein islands of KAC1 that reorganized and dissipated in the region of blue light.

**Supplemental Movie S4.** Transient expression of 35S_pro_:YFP:KAC1 in *N. benthamiana* treated with 10 µM Latrunculin B blocked the major reorganization of the KAC1 islands exposed to regions of blue light (rectangle).

**Supplemental Movie S5.** Transient expression of 35S_pro_:PMI1:YFP in *N. benthamiana* revealed a F-actin localized dark-state and swift dissipation of PMI1 when exposed to a region of blue light (rectangle).

**Supplemental Movie S6.** Transient expression of 35S_pro_:PMI1:YFP in *N. benthamiana* treated with 10 µM Latrunculin B did not interfere with the blue light-induced dissipation of PMI1 when exposed to a region of blue light (rectangle).

**Supplemental Movie S7.** Stable expression of 35S_pro_:PMI1:YFP in the *phot1 phot2* double mutant background blocked the dissipation of PMI1 in response to a region of blue light (rectangle).

**Supplemental Movie S8.** Transient expression of 35S_pro_:PMI1:YFP in *N. benthamiana* exposed to a perimeter of blue light stimuli (rectangles) revealed lateral movement of PMI1 that was corralled by the blue light perimeter.

**Supplemental Movie S9.** Transient expression of 35S_pro_:PMI1:YFP in *N. benthamiana* treated with 10 µM Latrunculin B and exposed to a perimeter of blue light stimuli (rectangles) did not block the corralling effect of PMI1 with the blue light perimeter.

**Supplemental Data Set 1.** Combined replicate list of mass spectrometry results featuring protein candidates and their associated peptide counts and sequence coverage.

## Supplementary Material

kiac193_Supplementary_Data

## Notes

M.E.D. designed the experiments with guidance from R.P.H. M.E.D. performed the experiments and analyzed the data. M.E.D. and R.P.H. interpreted the data. M.E.D. wrote the manuscript and R.P.H. edited the manuscript.

The author responsible for distribution of materials integral to the findings presented in this article in accordance with the policy described in the Instructions for Authors (https://academic.oup.com/plphys/pages/general-instructions) is: Roger P. Hangarter (rhangart@indiana.edu).
