# Supplementary material for: Light-induced displacement of PLASTID MOVEMENT IMPAIRED1 precedes light-dependent chloroplast movements
Source: Plant Physiol. 2022 Apr 27;189(3):1866–80. doi: 10.1093/plphys/kiac193 (PMC9237684; doi:10.1093/plphys/kiac193)
Supplement: kiac193_Supplementary_Data [file kiac193_supplementary_data.zip › Supplemental Figures Combined New.pdf]

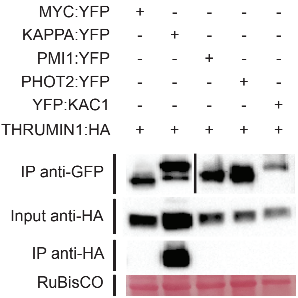


**Supplemental Figure S1. THRUMIN1 does not directly associate with Phot2, PMI1, and KAC1.** THRUMIN1:HA was co-transiently expressed in *N. benthamiana* leaf cells with either PMI1:YFP, Phot2:YFP, or YFP:KAC1 with KAPPA:YFP serving as a positive control and MYC:YFP as a negative control. Only KAPPA:YFP co-immunoprecipitated with THRUMIN1:HA. Protein bands do not represent true protein size since the blots were stitched together. Similar results were obtained in at least 3 independent experiments. This figure supports Table 1 in the main text.

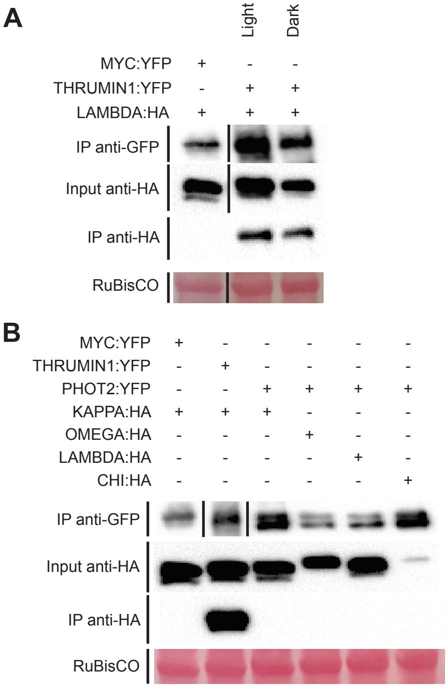


**Supplemental Figure S2. 14-3-3 LAMBDA associates with THRUMIN1 regardless of light treatment, but not Phot2.** (A) 35Spro:THRUMIN1:YFP was co-transiently expressed with 35Spro:LAMBDA:HA in *N. benthamiana* leaf cells and the plants were subjected to either 10 minutes of high blue light (~50 μmol/m2/s) or darkness before extraction. Regardless of light treatment, LAMBDA co-immunoprecipitated with THRUMIN1. (B) 35Spro:Phot2:YFP was co-transiently expressed with either 35Spro:KAPPA:HA, 35Spro:OMEGA:HA, 35Spro:LAMBDA:HA, or 35Spro:CHI:HA in *N. benthamiana* leaf cells. Based on the 35Spro:THRUMIN1:YFP/35Spro:KAPPA:HA association as a positive control, Phot2 did not associate with any of the 14-3-3 proteins tested. Similar results were obtained in at least 3 independent experiments. 35Spro:MYC:YFP was used as a negative control for all experiments and protein samples were extracted 48 hours post-infiltration. Ponceau-S stain was used as a loading control for total protein as demonstrated by RuBisCO. Protein bands do not represent true protein size since the blots were stitched together. This figure supports Table 1, Fig.1, and Fig.3 in the main text.

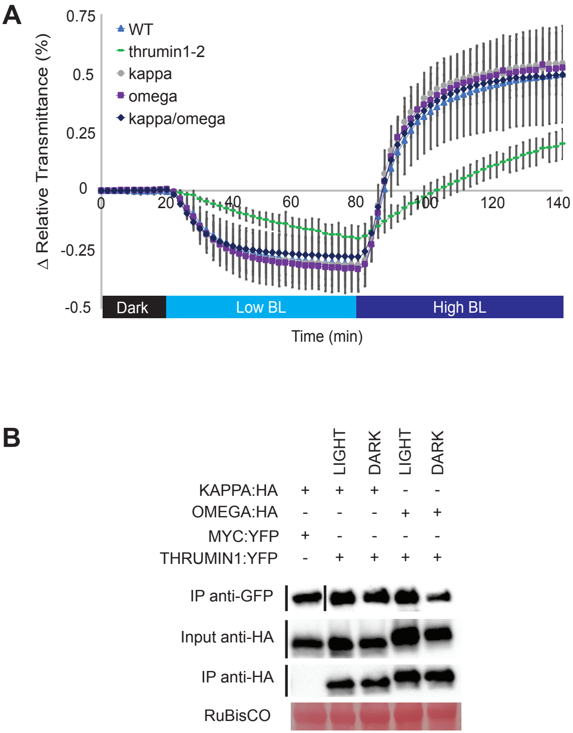


**Supplemental Figure S3. THRUMIN1 associates with 14-3-3 KAPPA and OMEGA independent of blue light (BL) stimulus but kappa and omega mutants have normal chloroplast movements.** (A) Leaf light transmittance assays with Col-0 wild type, *thrumin1-2* mutant, *kappa* mutant, *omega* mutant, and *kappa omega* double mutant demonstrated normal chloroplast movements. Error bars represent the standard deviation in transmittance values for 8-12 individual plants per genotype. The leaf transmittance assay was repeated 3 independent times for technical replicates. (B) Both 35Spro:KAPPA:HA and 35Spro:OMEGA:HA co-immunoprecipitated with 35Spro:THRUMIN1:YFP when co-transiently expressed in *N. benthamiana* regardless of light treatment. Plants were subjected to either 10 minutes of high blue light (~50 µmol/m2/s) or darkness before extraction. Similar results were obtained in at least 3 independent experiments. 35Spro:MYC:YFP was used as a negative control for all experiments and protein samples were extracted 48 hours post-infiltration. Ponceau-S stain was used as a loading control for total protein as demonstrated by RuBisCO. Protein bands do not represent true protein size since the blots were stitched together. This figure supports Table 1, Fig.1, and Fig.3 in the main text.

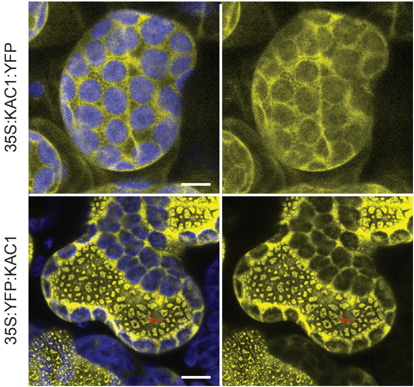


**Supplemental Figure S4. Arrangement of the YFP fusion protein affects the localization of KAC1.** 35Spro:KAC1:YFP was transiently expressed in *N. benthamiana* and conferred cytoplasmic localization whereas the 35Spro:YFP:KAC1 transgene conferred localization to distinct clusters (red arrows) in regions surrounding the chloroplasts. Similar results were obtained in at least 3 independent experiments. Chlorophyll autofluorescence is false-colored blue and the YFP channel is false-colored yellow. Scale bar = 5 μm. This figure supports Fig.2 in the main text and the YFP:KAC1 images are duplicates.

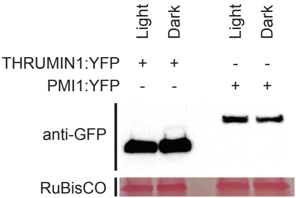


**Supplemental Figure S5. The PMI1 relocalization from blue light-irradiated regions of the cell is not due to protein degradation.** 35Spro:PMI1:YFP was transiently expressed in *N. benthamiana* leaf cells and the plants were subjected to either 10 minutes of high blue light (~50 µmol/m2/s) or darkness before extraction. 35Spro:THRUMIN1:YFP was used as a control. There was no detectable difference in PMI1 protein abundance in the light versus the dark detected by immunoblot imaging. Similar results were obtained in at least 3 independent experiments. Protein samples were extracted 48 hours post-infiltration. Ponceau-S stain was used as a loading control for total protein as demonstrated by RuBisCO. This figure supports Fig.4 and Fig.5 in the main text.

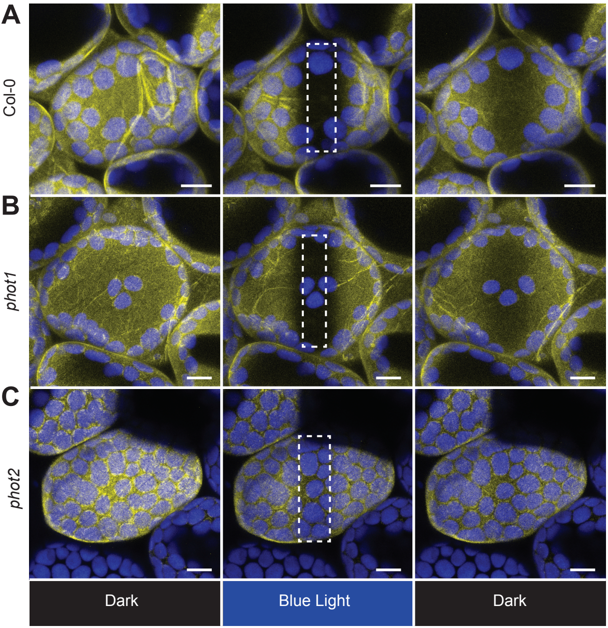


**Supplemental Figure S6. Phototropin2 is the main photoreceptor initiating the relocalization of PMI1 in high blue light.** 35Spro:PMI1:YFP was stably expressed in (A) Col-0, (B) phot1, and (C) phot2 backgrounds. In response to stimulation with a region of high blue light (white rectangle) the phot1 mutant background showed a full PMI1 relocalization response while the phot2 background showed minimal relocalization compared to Col-0. Representative frames of dark (514nm), blue light (470nm, white rectangle), and post-blue light darkness were selected (Note that the duration of the high blue light treatment was not long enough to result in significant chloroplast movements). Similar results were obtained in at least 3 independent experiments. Chlorophyll autofluorescence is false-colored blue and the YFP channel is false-colored yellow. Scale bar = 5 µm. This figure supports Fig.4 in the main text.
