# Supplementary material for: Light-induced displacement of PLASTID MOVEMENT IMPAIRED1 precedes light-dependent chloroplast movements
Source: Plant Physiol. 2022 Apr 27;189(3):1866–80. doi: 10.1093/plphys/kiac193 (PMC9237684; doi:10.1093/plphys/kiac193)
Supplement: kiac193_Supplementary_Data [file kiac193_supplementary_data.zip › Supplemental Movie Legends.pdf]

Supplemental Movie S1

$35S_{pro}$:THRUMIN1:YFP expressed in the Col-0 wild type background with whole-field blue light (470 nm) treatment demonstrated the biased reorganization of the THRUMIN1-cp-actin filaments on the leading edge of the chloroplast during movement.

Supplemental Movie S2 $35S_{pro}$:THRUMIN1:YFP expressed in the *kac1-3 kac2-1* double mutant background with whole-field blue light (470 nm) treatment elicited a robust hyper-localization of THRUMIN1 to the cp-actin and cortical actin filaments.

Supplemental Movie S3

Transient expression of $35S_{pro}$:YFP:KAC1 in *N. benthamiana* exposed to regions of blue light (rectangles) demonstrated protein islands of KAC1 that reorganized and dissipated in the region of blue light.

Supplemental Movie S4

Transient expression of $35S_{pro}$:YFP:KAC1 in *N. benthamiana* treated with 10μM Latrunculin B blocked the major reorganization of the KAC1 islands exposed to regions of blue light (rectangle).

Supplemental Movie S5

Transient expression of $35S_{pro}$:PMI1:YFP in *N. benthamiana* revealed a F-actin localized dark-state and swift dissipation of PMI1 when exposed to a region of blue light (rectangle).

Supplemental Movie S6

Transient expression of $35S_{pro}$:PMI1:YFP in *N. benthamiana* treated with 10μM

Latrunculin B did not interfere with the blue light-induced dissipation of PMI1 when exposed to a region of blue light (rectangle).

Supplemental Movie S7

Stable expression of $35S_{pro}$:PMI1:YFP in the *phot1 phot2* double mutant background blocked the dissipation of PMI1 in response to a region of blue light (rectangle).

Supplemental Movie S8

Transient expression of $35S_{pro}$:PMI1:YFP in *N. benthamiana* exposed to a perimeter of blue light stimuli (rectangles) revealed lateral movement of PMI1 that was corralled by the blue light perimeter.

Supplemental Movie S9

Transient expression of $35S_{pro}$:PMI1:YFP in *N. benthamiana* treated with 10μM Latrunculin B and exposed to a perimeter of blue light stimuli (rectangles) did not block the corralling effect of PMI1 with the blue light perimeter.
